# Signal detection shapes ornament allometry in functionally convergent Caribbean *Anolis* and Southeast Asian *Draco* lizards

**DOI:** 10.1111/jeb.14102

**Published:** 2022-09-30

**Authors:** Thomas C. Summers, Terry J. Ord

**Affiliations:** ^1^ Evolution and Ecology Research Centre, and the School of Biological, Earth and Environmental Sciences University of New South Wales Kensington New South Wales Australia

**Keywords:** constraint, dewlap, environment, light, sexual selection, visual noise

## Abstract

Visual ornaments have long been assumed to evolve hyper‐allometry as an outcome of sexual selection. Yet growing evidence suggests many sexually selected morphologies can exhibit other scaling patterns with body size, including hypo‐allometry. The large conspicuous throat fan, or dewlap, of arboreal Caribbean *Anolis* lizards was one ornament previously thought to conform to the classical expectation of hyper‐allometry. We re‐evaluated this classic example alongside a second arboreal group of lizards that has also independently evolved a functionally equivalent dewlap, the Southeast Asian *Draco* lizards. Across multiple closely related species in both genera, the *Anolis* and *Draco* dewlaps were either isometric or had hypo‐allometric scaling patterns. In the case of the *Anolis* dewlap, variation in dewlap allometry was predicted by the distance of conspecifics and the light environment in which the dewlap was typically viewed. Signal efficacy, therefore, appears to have driven the evolution of hypo‐allometry in what was originally thought to be a sexually selected ornament with hyper‐allometry. Our findings suggest that other elaborate morphological structures used in social communication might similarly exhibit isometric or hypo‐allometric scaling patterns because of environmental constraints on signal detection.

## Graphical abstract

The large conspicuous dewlap of anoles and other lizards that were previously thought to be hyper‐allometric and the product of sexual selection are in fact isometric or hypo‐allometric and the product of signal detection.

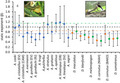

## 1 INTRODUCTION

Sexual selection is considered to be the primary driving force behind the evolution of exaggerated size in ornaments and weapons among species. When these structures honestly reflect individual condition, the size of ornaments or weapons can operate as a condition‐dependent signal that receivers can use to assess the relative quality or fighting potential of its owner (review by Andersson, 1982, 1994; Bonduriansky & Rowe, 2005; Rowe & Houle, 1996).

Examples of morphological structures assessed by conspecifics as condition‐dependent signals include exaggerated plumage in birds (Jarvisto et al., 2013; McFarlane et al., 2010; Pryke & Andersson, 2005; van Dongen & Mulder, 2007), enlarged fins and head crests in fish (Benson & Basolo, 2006; Karino et al., 2011; Locatello et al., 2012; Ord & Hsieh, 2011) and protruding eyestalks among stalk‐eyed flies (Cotton et al., 2004; Hingle et al., 2001; Wilkinson et al., 1998). These sexual display structures generally operate to advertize individual condition to potential mates or as the focal point of territorial displays as a means of intimidating and deterring rivals from physical combat (Andersson & Iwasa, 1996; Arnott & Elwood, 2009; Iwasa & Pomiankowski, 1994). While the overall size of these structures can reflect the intensity of sexual selection operating within species (see Andersson & Simmons, 2006; Kuijper et al., 2012; Tazzyman et al., 2014), much less is known about how the environment—specifically the conditions in which signals are viewed—might also influence the exaggeration of species morphological structures.

If these structures are honest indicators of condition, selection is likely to favour individuals who develop increasingly large costly visual signals. In this scenario, only larger individuals within populations are able to invest in exaggerating the apparent size of their display structures (Emlen et al., 2012; Grafen, 1990; Kotiaho, 2001; Kotiaho et al., 1998; Nur & Hasson, 1984). In turn, these individuals are afforded fitness benefits for advertising their disproportionately superior condition towards potential mates and territorial rivals (Biernaskie et al., 2014; Bonduriansky & Day, 2003).

This relationship between disproportionate sexual signal size and its correlated fitness advantages underlies the theory of hyper‐allometry in sexually selected morphologies (Cheverud, 1982; Lande, 1985). It argues the size of exaggerated structures used in mating or territorial displays should exhibit a particular scaling relationship with body size within populations or species (Cock, 1966; Gould, 1966; Green, 1992; Huxley, 1924, 1932; Petrie, 1992). That is, the allometric scaling of this relationship should reflect a power function with an exponent greater than 1 (i.e. X^*b*^ where *b* > 1) termed ‘hyper‐allometry’. This is because larger individuals are able to invest more in exaggerated sexual characteristics than smaller individuals, resulting in a disproportionate increase in the size of ornaments or weapons with increasing body size across males within the population. Furthermore, the magnitude that exponents exceed 1 (isometry; i.e. a one‐to‐one proportional increase in the size of a sexual signal with increases in body size) has been argued to reflect the extent to which the exaggerated structure is targeted by sexual selection (Green, 1992, 2000; Petrie, 1992; Kodric‐Brown et al., 2006).

There are many instances where sexual display structures exhibit hyper‐allometric relationships (*b* > 1; e.g. Cuervo & Moller, 2009; Eberhard, 2002a, 2002b; Kodric‐Brown et al., 2006; Outomuro & Cordero‐Rivera, 2012; Petrie, 1988; Watkins, 1998), but recent work suggests hyper‐allometry in sexually selected morphologies are not as common as previously thought (e.g. Bertin & Fairbairn, 2007; Briceno et al., 2005; Cuervo & Moller, 2001; Eberhard, 2002a, 2002b; Egset et al., 2011). Furthermore, there is theoretical evidence that implies selection could just as easily favour sexual display structures that are isometric (exponents equalling 1) or even hypo‐allometric (exponents less than 1; see Bonduriansky & Day, 2003; reviewed by Bonduriansky, 2007 and Voje, 2016).

Species might be prevented from evolving disproportionately large sexual display structures—and its associated exponent *b* > 1—in a number of ways. For example, intrinsic factors regulate the developmental relationship between body size and morphological characteristics among species that can affect the extent those characteristics exhibit hyper‐allometry (e.g. Bolstad et al., 2015; Egset et al., 2011, 2012; Pelabon et al., 2013; Tobler & Nijhout, 2010). What is less understood is how extrinsic factors, such as local environmental conditions, might impact the allometric exponents of sexually selected morphology. For example, individuals with larger display structures are generally exposed to greater predation (Andersson, 1982, Klomp et al., 2016; see also Kotiaho, 2001; Zuk & Kolluru, 1998) or suffer increased constraints on locomotion (Barbosa & Moller, 1999; Basolo & Alcaraz, 2003; Swallow et al., 2000). These costs can place an upper limit on ornament or weapon size to ultimately reduce the allometric exponents of display structures across males (Summers & Ord, 2022). However, few investigations have measured the extent to which the viewing conditions in which ornaments and other display structures are evaluated might shape the allometric relationships of those structures. In particular, the size of structures used in sexual communication is likely to play a role in the detection of those structures by receivers and this will, in turn, be influenced by the physical properties of the environment.

The viewing conditions imposed by the surrounding environment will impact how well display structures are detected and processed by their intended targets (e.g. Menezes & Santos, 2020; Milinski & Bakker, 1990; Ord et al., 2007; Ord & Stamps, 2008; Peters et al., 2007; see also Endler, 1992, 1993; Fleishman, 1992; Hebets & Papaj, 2005; Wiley, 2006). In relatively open, bright habitats, display structures can be readily detected and their size assessed by conspecifics over longer distances compared to closed, darker habitats. This will be further aggravated by increasing visual noise from windblown vegetation (Peters et al., 2008), where distant receivers are more likely to have difficulty in assessing the size of (or even detecting) a display structure (Endler, 1992, 1993; Wiley, 2017). The need for improved detection when viewing conditions are difficult would presumably promote the evolution of large sexually selected and condition‐dependent structures. That is, both the need for improved signal detection and sexual selection should work in tandem to produce visual signals that exhibit a pattern of increasingly disproportionate size with increasing body size (i.e. hyper‐allometry).

However, the ability of receivers to discriminate differences in object size decreases with the absolute size of the stimulus (i.e. Weber's Law; Akre et al., 2011; Akre & Johnsen, 2016; Eberhard et al., 2018). In other words, as a signal structure becomes larger, the benefits it might achieve by being more detectible eventually decrease such that there are diminishing returns for every incremental increase in a sexual structure's size. This means the biggest individuals in the population would receive little additional benefit from exaggerating a signal beyond a particular size, because it is less likely to further enhance their detection as receivers are less likely to perceive the difference in size (e.g. Akre et al., 2011). Here, the allometry of these sexual structures might be expected to be isometric, if not hypo‐allometric. Several studies have reported hypo‐allometric patterns in morphological weapons within species of some stag beetles (i.e. ‘static’ allometry in which allometry is estimated across individuals; Knell et al., 2004) or among species of cervids and bovids (‘evolutionary’ allometry where scaling patterns are estimated across the mean values of species; Lemaitre et al., 2014; Tidiere et al., 2017). These patterns were reasonably attributed to developmental costs that effectively cap the maximum size weapons can reach. However, there have yet to be any substantial attempts to empirically determine the extent to which signal detection might contribute towards allometric scaling patterns in sexual display structures (but see Lopez‐Palafox et al., 2020 for a rare example). Doing so could potentially reveal why some ornaments or structures used in sexual display exhibit different allometric relationships across closely related taxa (e.g. because those taxa optimize signal efficacies that depend on different habitats).

In this study, we examined the allometry of a conspicuous display structure in a group of closely related arboreal *Anolis* lizards (family: Iguanidae) from the islands of Puerto Rico and Jamaica. Males use a conspicuous throat fan, or dewlap, as part of a visual display used to establish and maintain territories that encompass several trees (Jenssen et al., 2000; Nicholson et al., 2007; Ord, 2021; Ord et al., 2007; Ord & Stamps, 2008). The size of this dewlap in males has previously been shown to be positively correlated with bite force within species (Lailvaux & Irschick, 2007; Vanhooydonck et al., 2005) and has also been shown to be correlated with indices of sexual size dimorphism across species (Fitch & Hillis, 1984; Vanhooydonck et al., 2015). This implies male dewlap size could provide an honest indicator of potential fighting ability and reflect differences in the intensity of competition for territories among species. Dewlap size has also been classically reported to exhibit hyper‐allometry within species (Echelle et al., 1978; see also Kodric‐Brown et al., 2006; Petelo & Swierk, 2017), which has led to the general view that the exaggerated size of *Anolis* dewlaps is the product of sexual selection. In particular, the *Anolis* dewlap has been explicitly used to validate that hyper‐allometric exponents can be interpreted as the product of sexual selection (see Kodric‐Brown et al., 2006). Yet the size of the dewlap has also been demonstrated to play a critical role in the detection of *Anolis* territorial displays (Macedonia et al., 2003; Ord et al., 2007; Ord & Stamps, 2008). There is also evidence to suggest that the dewlap has little impact on the ability of males to obtain or defend territories or acquire mates (Tokarz, 2002; Tokarz et al., 2003, 2005), which questions the function of the dewlap as a condition‐dependent signal.

On the opposite side of the world to the Caribbean *Anolis* lizards are the Southeast Asian *Draco* lizards (family: Agamidae), another arboreal genus that are remarkable parallels in behaviour and ecology to the *Anolis* (Ord & Klomp, 2014; Ord, Klomp, et al., 2021a). In particular, *Anolis* and *Draco* have convergently evolved a similar exaggerated dewlap as part of an overall territorial display (Klomp et al., 2016; Mori & Hikida, 1993; Ord et al., 2015). These displays advertize the ownership of territories that include several trees in a similar manner to the *Anolis* (Ord, 2021; Ord, Klomp, et al., 2021a). Furthermore, the size of the dewlap in male *Draco* lizards also seems to play an important role in signal detection in the same way that it does in *Anolis* (Klomp et al., 2016). Both *Anolis* and *Draco* lizards occupy a range of remarkably similar habitats (Ord, Klomp, et al., 2021a). We leveraged the convergent and functionally equivalent evolution of the dewlap in *Anolis* and *Draco* lizards to provide a comparative investigation into the allometry of a morphological structure previously used to highlight the allometry of sexual selection (at least in the case of *Anolis*; see Kodric‐Brown et al., 2006). By focusing on species occupying a range of environments, we sought to examine the extent to which signal detection might impact the nature of allometric scaling relationships among these species.

## 2 MATERIALS AND METHODS

We began our investigation by first determining whether the allometries of the convergently evolved *Anolis* and *Draco* dewlaps conformed to the original predictions established by Kodric‐Brown et al. (2006): exaggerated structures targeted by sexual selection within species—including the *Anolis* dewlap specifically—should consistently exhibit hyper‐allometry and explicitly exponents 1.5 or greater. To accomplish this, we extracted images of the dewlap fully extended by free‐living male *Anolis* and *Draco* lizards in videos of territorial advertisement displays. From these images, we estimated dewlap area and body length (snout‐to‐vent length, SVL; see Supporting Information) for each male lizard to compute within‐taxon static allometries (see Section 2.2).

No *Anolis* or *Draco* species was found to exhibit hyper‐allometry in dewlap size. There was, however, considerable variation among taxa in computed allometric exponents, ranging from isometry to hypo‐allometry in both genera. We then evaluated the extent to which this variation in dewlap allometry was explained by differences in the environmental conditions experienced by species that are known to affect signal detection: visual noise from windblown vegetation (Ord et al., 2007), ambient light (Ord et al., 2013), and the distance over which displays would be typically detected by territorial neighbours (receiver distance; Ord, 2012).

### 2.1 Data collection

We studied multiple free‐living territorial males of *Anolis* (median males examined per taxon = 21 range: 13–27) from 14 taxa (11 species including six geographically separated population pairs within three species) from Puerto Rico and Jamaica, and multiple free‐living territorial males of *Draco* (median males examined per taxon = 13; range: 9–19) from six taxa (five species including two geographically separated populations from one species) from peninsula Malaysia and Borneo. See Tables S1–S3 for location details and taxon sample sizes.

Males in both genera establish territories that typically comprise several trees or shrubs and frequently advertize territory ownership from arboreal perches using elaborate visual displays. These displays include the extension of a large, often colourful dewlap, with males typically orienting themselves on perches in order to maximize the conspicuousness of displays to a variety of surrounding conspecific receivers (TJO personal observation; see also Klomp et al., 2017). These receivers include resident females and territorial male neighbours viewing displays over a range of distances (Ord, 2012).

Protocols used to observe lizards, video recording of behaviour and the collection of data on environmental conditions were identical for both *Anolis* and *Draco* and are outlined elsewhere (Ord, Klomp, et al., 2021a). Briefly, lizards were spotted by slowly walking through the environment and recorded using a digital video camcorder positioned on a tripod several meters away from the animal. Video recording typically lasted for 10–30 min during which time the lizard was kept in profile to the camcorder by slowly shifting the tripod whenever the lizard moved position. At the end of the recording session, a Ping‐Pong ball of known size was video recorded at each site to calibrate the distance of the camcorder from the lizard (see Ord et al., 2007). This was used to convert estimates of dewlap area and body size from pixels to millimetres. This calibration protocol was also important for the motion analysis of windblown vegetation captured in the backgrounds of video recordings. These analyses of visual noise were conducted as part of earlier studies (e.g. see Ord et al., 2010; Ord, Klomp, et al., 2021a), but were on the same videos used here to obtain data on dewlap size and body length. Ambient habitat light was measured at the end of the recording session at the site of first display for each lizard using a LI‐190SA Quantum Sensor attached to a handheld LI‐250A light meter. Two measures approximating the position of the lizard's left and right eye were averaged to provide an estimate of the amount of ambient light entering the eye at a position typical of where lizards are found in the environment. Receiver distance was determined by the average distance to all adult male neighbours within the line of sight of the focal male lizard and was only obtained for *Anolis* because of the difficulty of obtaining comparable data for *Draco* lizards, who were generally harder to spot because of their more cryptic body patterning. The level of background motion from windblown vegetation, or visual ‘noise’, was estimated for both *Anolis* and *Draco* lizards using computational motion analysis of the backgrounds of display clips (Peters et al., 2002; full details are provided in Ord et al., 2007). These estimates of background visual noise consisted of the maximum speed of movement occurring in display backgrounds, averaged across all displays recorded for a given male.

From the archive library of video recordings, we exported still images during periods where males had their dewlap fully extended and without the dewlap extended in order to more accurately measure body length. The external anatomy of the *Draco* male dewlap was slightly different to that of *Anolis* by having an additional extension from the neck on either side of the dewlap—which we refer to as ‘lapels’—and we included both the dewlap and these lapels in estimates of area. The outlines of the dewlaps (and the lapels of male *Draco* lizards) were digitally measured from extracted video stills using software ImageJ v.142q (National Institutes of Health, http://rsbweb.nih.gov/nih‐image/). Lizard body length estimates, SVL, were based on a measure from the snout to the approximated vent position at the base of the tail. Three measures of dewlap area and SVL were taken from three separate bouts of display for each male lizard (repeatability estimates across the three measures are provided in Tables S1–S3). These three measurements were averaged for each male to obtain a single estimate of dewlap area, dewlap area inclusive of lapels (in the case of *Draco*) and body length. Averaging was done to control for potential measurement error. In the case of dewlap area, this ‘average’ still represented the size of the dewlap at its maximum extension (not its average extension length).

To confirm the accuracy of measurements taken from video stills, we used 15 robotic *Draco* lizards developed by Ord, Blazek, et al. (2021b) to compare estimates of dewlap area and body length taken from stills to their ‘true’ physical values (the dewlaps of these robots varied in size across a range comparable to adult males in life; data presented in Ord, Blazek, et al. (2021b)). These robots were programmed to perform the dewlap display of male *Draco sumatranus* and were positioned in the environment to mimic the typical habit of lizards in life. The robots were then video recorded using identical protocols to those used to record the behaviour of live lizards (described previously). A digital video camcorder mounted on a tripod was positioned several metres away from the displaying robot. A Ping‐Pong ball of known size was video recorded where the robot had been positioned to calibrate the distance of the camcorder from the robot. Stills of the robot with its dewlap at full extension were then extracted from the video, and ImageJ was used to measure both the area of the dewlap and length of the robot body. There was no statistical difference in either dewlap area or the length of the body compared to their known values (paired *t* test, dewlap area: d.f. = 14, *t* = −1.27, *p* = 0.23; body length: d.f. = 14, *t* = 0.37, *p* = 0.72; NB: a Pearson's correlation also confirms a strong, positive correlation between known values and those estimated from video: dewlap area: d.f. = 13, *r* = 0.62, *p* = 0.01; body length: d.f. = 13, *r* = 0.64, *p* = 0.01). Video estimates of dewlap area were on average of 3.6% smaller than known values, but not statistically different from zero with a 95% confidence range of 9.7% smaller to 2.5% bigger than known values. Video estimates of body length were on average of 0.5% longer than known values, but again this was not statistically different from zero with a 95% confidence range of 2.6% shorter to 3.7% longer than known values. These analyses show the measurement of dewlap area and body length from video stills of displaying free‐living lizards provide a reasonable and non‐invasive estimate of these features.

### 2.2 Data analysis

All statistical analyses were performed using R version 3.2.4 (R Development Core Team, The R Foundation for Statistical Computing, Vienna, Austria). Dewlap and lapel area measurements were first linearized using a square‐root transformation (Bonduriansky, 2007). All data was then natural‐log transformed to enable linear regressions to be applied that were statistically equivalent to a power function, in which the slope of the regression corresponded to the allometric exponent (*b*). Intercept values were not evaluated as variation among taxa in slope estimates make direct comparisons of intercept estimates difficult, unless body size data is subject to some form of mean centring transformation (see Bolstad et al., 2015; Enders & Tofighi, 2007; Tidiere et al., 2017; Voje & Hansen, 2013; White & Gould, 1965). Mean centring was problematic in our study because of non‐overlapping size ranges of several taxa for both *Anolis* and *Draco* (Figure S2). The use of intercept values computed from allometric analyses are discussed further below.

Our first set of analyses focussed on estimating the static allometric exponents of dewlap area within each *Anolis* and *Draco* taxon. Specifically, static allometries were computed across male lizards within each population (for those species with more than one studied population) and each species separately, for a total of 14 within‐taxon static allometries for *Anolis* and six within‐taxon static allometries for *Draco*. Our previous work on other animals has indicated that estimates of static allometry can be accurately computed when roughly 10 or more individuals have been sampled over a wide range of adult body sizes in a population (Summers & Ord, 2022). Furthermore, there was no association between the fit of computed static allometries and sample size (regression of *R* ^2^ as a function of *N*, *Anolis*: *F*_1,12_ = 0.03, *p* = 0.86; *Draco*: *F*_1,4_ = 0.06–0.13, *p* = 0.74–0.82; see also Tables S1–S3). That is, taxa with fewer males surveyed did not have poorer fitting allometric regressions. The distribution of body sizes for each taxa examined is provided in Figure S1 and shows a broad range of male body sizes were sampled for each taxa. We also confirmed that our sampling was not confounded by potential difficulties in locating lizards in low light environments (Poisson generalized linear model of sample size on ambient light, *Anolis*: *z* = −0.358, *p* = 0.720; *Draco*: *z* = −0.188, *p* = 0.851; Figure S1ii and iv) or for populations where lizards might have been more widely spatially distributed (Poisson‐generalized linear model of sample size on receiver distances, *Anolis* only: *z* = −0.769, *p* = 0.442; Figure S1iii).

There has been some debate over whether allometry should be calculated using ordinary least squares (OLS) or reduced major axis regressions, but a general consensus has begun to emerge that OLS regression provides a more reliable analysis for estimating allometry relationships (Hansen & Bartoszek, 2012; Kelly & Price, 2004; Kilmer & Rodriguez, 2017; Voje et al., 2014). Estimates of taxon allometries were, therefore, carried out on natural‐log transformed data using OLS regressions and the base function ‘lm’ in R.

Our second set of analyses examined the extent to which variation in dewlap allometry among taxa could be explained by the types of environments taxa occupied. Exponents (specifically the *b* estimate) were entered into a series of phylogenetic regressions with various combinations of ambient light, visual noise, receiver distance (in the exclusive case of *Anolis*) and their interactions. These analyses relied on an Ornstein–Uhlenbeck (OU) process to model the residual variation in regressions and were implemented with the package ‘phylolm’ version 2.6.2 (Ho & Ane, 2014) and the phylogeny developed by Nicholson et al. (2005) for *Anolis* and Ord et al. (2020) for *Draco*. Populations were positioned on these phylogenies based on the minimum intra‐island population divergence reported for Jamaican *Anolis* species by Jackman et al. (2002) and Philippine *Draco* species by McGuire and Heang (2001). Relative support for each phylogenetic regression model was evaluated using a sample size correction of the Akaike information criterion (AIC_c_). These values were converted into model weights (AIC_ω_ Burnham & Anderson, 2002) that compared proportional support of a given model in relation to all others applied. Computed *t*‐values for the included predictor variables were used to determine the magnitude and direction of statistical effects, with values *t* > 1.96 considered to be those that can be statistically distinguished from zero. An additional analysis was also conducted for *Draco* that replicated analyses based on an estimate of dewlap area inclusive of the lapel.

Our final set of analyses investigated the evolutionary allometry of the dewlap—differences in mean‐taxon dewlap area as a function of mean‐taxon body length—and the extent to which the average size of the dewlap across taxa could be predicted by the type of habitat occupied by species. First, we averaged the natural‐logged transformed data across males within a given taxon to compute the mean size of the dewlap and mean body length of that taxon. Second, evolutionary exponents (i.e. the slope, *b*) were calculated using a phylogenetic OU regression of mean‐taxon dewlap area on mean‐taxon body length separately for *Anolis* and *Draco*. An additional analysis was also conducted for *Draco* that used an estimate of dewlap area inclusive of the lapel. Third, in order to evaluate the extent to which habitat accounted for differences in mean area of the dewlap (relative to taxon body length), we applied an additional set of regressions that included various combinations of ambient light, visual noise, receiver distance (in the case of *Anolis*) and their various interactions. In these analyses, we used the residuals of mean‐taxon dewlap area as a function of mean‐taxon body length computed from the bivariate evolutionary allometry regressions to focus the analyses specifically on mean‐taxon dewlap area controlling for taxon body size. An alternative approach of standardizing dewlap area would be to use the intercept values computed from taxon static allometries in which body size is centred on a common value for all species. This approach is generally not appropriate for our study because there are several species of *Anolis* and *Draco* that do not overlap in size range (i.e. no common size value exists for all taxa). It is still possible to compute a global mean for all species of a genus and then rely on intercept values that might be inferred beyond the range of the available data for that species. There are statistical concerns with doing this, but we nevertheless repeat the habitat analyses in which residual dewlap areas are substituted with static allometry intercept values where body size has been centred on a global mean. These analyses are reported in Table S3 and are consistent with findings from habitat analyses of residual dewlap area and are not discussed further.

Note, estimates of ambient light for *Anolis evermanni* were known to be unusual because of the way in which this species was sampled, meaning this was unlikely to provide a true reflection of the light environment occupied by the species. We, therefore, adopted the approach of earlier studies (Ord et al., 2010) of re‐running analyses without *A. evermanni* when evaluating the relationship of habitat light. Analyses that include *A. evermanni* are provided in the Supporting Information.

## 3 RESULTS

### 3.1 Static allometry

**FIGURE 1:**
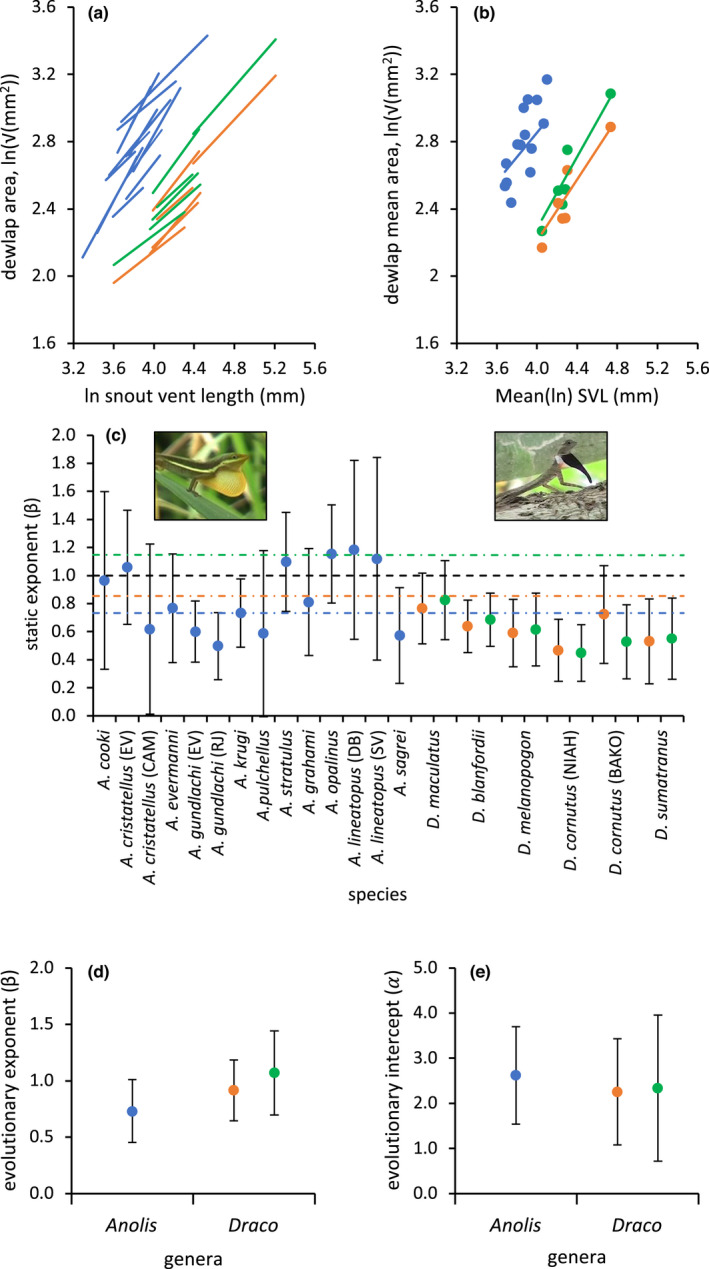
Allometries of *Anolis* dewlaps (blue), *Draco* dewlaps (orange) and *Draco* dewlaps + lapels (green). Shown in (a) are static allometric exponents for the 14 *Anolis* and 6 *Draco* taxa. Also shown are the evolutionary exponents (b) with corresponding taxon mean areas (mm^2^). Panel (c) shows static exponents for *Anolis* and *Draco* dewlaps compared to isometry (black dashed line). The blue dashed line is the *Anolis* dewlap evolutionary exponent, while the orange and green dashed lines are the *Draco* dewlap and dewlap + lapel evolutionary exponents, respectively. The images are stills taken from video that depict *A. krugi* and *D. melanopogon* male lizards with their dewlaps fully extended. The bottom panels show evolutionary exponents (d) and intercepts (e) for *Anolis* and *Draco*. All error bars are 95% confidence intervals.

No *Anolis* or *Draco* taxa exhibited hyper‐allometry in dewlap size (Figure 1c). Instead, allometric exponents of the dewlap for male *Anolis* lizards were on average of 0.84 with a 95% confidence interval (CI) of 0.77–0.91, and for male *Draco* lizards were on average of 0.62, with a 95% CI of 0.51–0.73 (in *Draco*, there was very little difference when dewlap areas included the lapels: 0.61 with a 95% CI of 0.49–0.73). That is, dewlap size in both genera exhibited hypo‐allometries, with larger male lizards possessing disproportionately *smaller* dewlaps compared to smaller male lizards.

**FIGURE 2:**
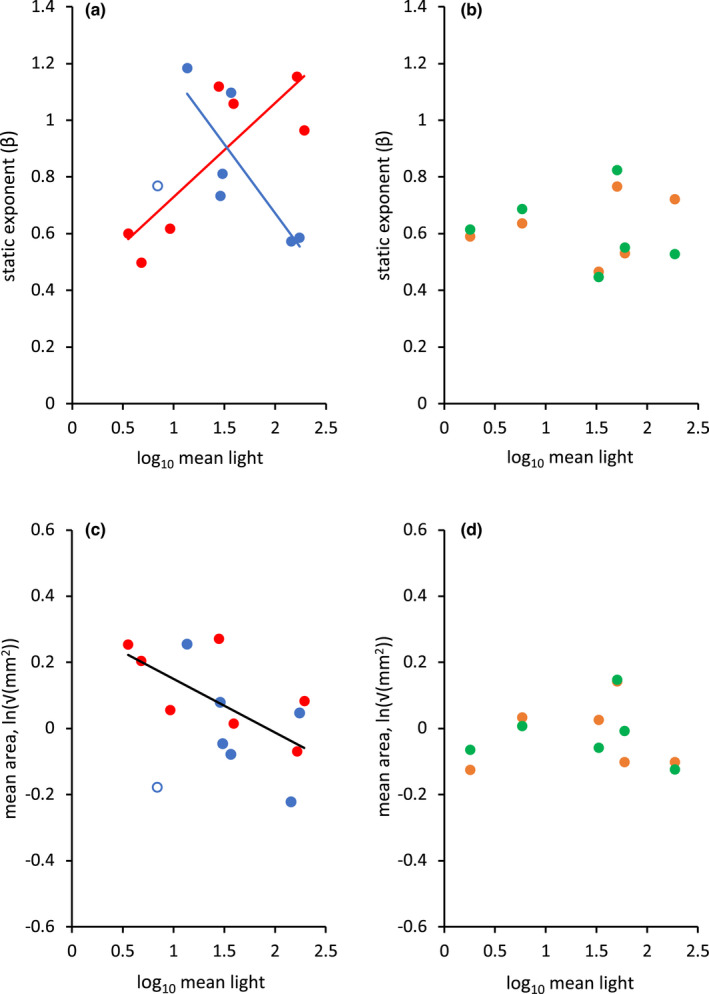
OU regressions of OLS static exponents and mean areas (mm^2^) with ambient light (log_10_). In panel (a) *Anolis* taxon exponents with distant signal receivers are shown in red, while those with close receivers are shown in blue (open blue circle refers to *A. evermanni* omitted from static exponent and mean area (mm^2^) analyses; see Section 2). Red and blue lines represent supported interaction between distant and close taxon exponents and their opposing trends with respect to increasing ambient light levels (see Table 1a). This is compared to (b) *Draco* dewlap (orange) and dewlap + lapel (green) exponents that exhibited no apparent correlation with light or any other environmental variable. The weak negative association of *Anolis* dewlap taxon mean areas (mm^2^) with ambient light is shown in (c). No such pattern was found among (d) *Draco* dewlap (orange) and dewlap + lapel (green) taxon mean areas (mm^2^).

There was, however, considerable variation among *Anolis* and *Draco* taxa in estimated allometric exponents (Figure 1c). In the case of *Anolis*, much of this variation appeared to be explained by an interaction between habitat light and receiver distance (Table 1a). Specifically, *Anolis* lizards communicating over increasingly longer distances in darker environments exhibited shallower dewlap static exponents (Figure 2a), whereas lizards communicating over shorter distances in brighter habitats exhibited steeper (although still hypo‐) static exponents (Figure 2a; NB: ambient light did not predict estimates of receiver distance—linear regression: *F*_1,12_ = 0.128, *p* = 0.726, Figure S1—which might have occurred if distant neighbouring lizards were harder to spot by the researcher in dimly lit environments). In contrast, there was no obvious environmental explainer of the differences in static exponents for the *Draco* dewlap (with or without inclusion of the lapel). However, we lacked information on receiver distance for *Draco* lizards and our sample size was also lower for *Draco* than *Anolis*. Furthermore, there was generally little variation in computed exponents across *Draco* taxa.

**TABLE 1:** Phylogenetic regressions of (a) *Anolis* dewlap, (b) *Draco* dewlap and (c) *Draco* dewlap + lapel OLS static allometric exponents (*b*) ranking all possible combinations of three key environmental variables: L, ambient light (log_10_); VN, visual noise; and RD, receiver distance

Exponent model	AIC_c_	∆AIC	AIC_ω_	Effect size							
				*t*‐light	*t*‐noise	*t*‐distance	*t*‐light × noise	*t*‐light × distance	*t*‐distance × noise	α	σ^2^
(a) *Anolis* (excluding *A. evermanni*) *N*_male lizards, taxa_ = 265, 13											
RD × L	6.92	0.00	0.77	−6.91		−8.49		8.23		<0.001	<0.001
L	11.35	4.42	0.08								
RD	12.01	5.09	0.06								
VN	12.23	5.31	0.05								
RD + L + VN + RD × L + L × VN	15.10	8.18	0.01								
RD + L	16.53	9.61	0.01								
L + VN	16.61	9.68	0.01								
RD + L + VN + RD × L	17.19	10.27	0.00								
RD + VN	17.40	10.48	0.00								
RD × VN	20.20	13.28	0.00								
L × VN	21.72	14.80	0.00								
RD + L + VN	23.72	16.79	0.00								
RD + L + VN + RD × VN	30.27	23.35	0.00								
RD + L + VN + L × VN	31.94	25.01	0.00								
RD + L + VN + RD × L + RD × VN + L × VN	54.70	47.78	0.00								
(b) *Draco* (dewlap) *N*_male lizards, taxa_ = 80, 6											
VN	−3.23	0.00	0.45		0.87					1.84	0.03
L × VN	−3.16	0.07	0.44	−1.12	−0.55		2.04			<0.001	<0.001
L	0.45	3.68	0.07								
L + VN	1.58	4.81	0.04								
(c) *Draco* (dewlap + lapel) *N*_male lizards, taxa_ = 80, 6											
L	−4.60	0.00	0.41	2.61						<0.001	<0.001
VN	−4.39	0.21	0.37		2.54					<0.001	<0.001
L + VN	−2.75	1.85	0.16	0.43	0.28					<0.001	<0.001
L × VN	−0.97	3.64	0.07								

### 3.2 Evolutionary allometry

The average size of the *Anolis* dewlap across taxa (16.79 mm^2^, 95% CI = 16.52–17.05) was generally larger than *Draco* (12.15 mm^2^, 95% CI = 11.55–12.74), but there was a high degree of overlap in the range of dewlap sizes between the two groups, particularly when comparing dewlap sizes of *Draco* that included the area of the lapel (Figure 1b; NB: average size of *Draco* dewlap inclusive of the lapel was 13.88 mm^2^ with a 95% CI of 13.20‐14.57). Furthermore, the evolutionary allometric exponent (Figure 1d) of the mean‐taxon dewlap size on mean‐taxon body length was similar, and largely hypo‐allometric, in both *Anolis* and *Draco*: 0.73 (95% CIs = 0.45–1.01) across *Anolis* taxa versus 0.92 (95% CIs = 0.65–1.19) across *Draco* taxa (or 1.07, 95% CIs = 0.70–1.44 based on dewlap size inclusive of the lapel). Similarly, estimates of the evolutionary intercept (Figure 1e) indicated both the *Anolis* (2.62, 95% CIs = 1.54–3.70) and *Draco* (2.25, 95% CIs = 1.08–3.43; with lapel: 2.34, 95% CIs = 0.72–3.96) were comparable, which again implies the dewlap was approximately similar in size between the two genera.

Finally, in the case of *Anolis* lizards, variation among taxa in the gross (taxon mean) size of the dewlap was negatively correlated with habitat light (Table 2a; Figure 2c). That is, *Anolis* taxa living in dimly lit environments, on average (and controlling for taxon body size), possessed larger dewlaps than taxa living in well‐lit environments. In the case of *Draco* lizards, variation in gross dewlap size could not be convincingly attributed to any environmental variable (*t* < 1.96; Table 2b,c; Figure 2d).

**TABLE 2:** Phylogenetic regressions of taxon (a) *Anolis* dewlap, (b) *Draco* dewlap and (c) *Draco* dewlap + lapel mean areas (mm^2^) ranking all possible combinations of three key environmental variables: L, ambient light (log_10_); VN, visual noise; and RD, receiver distance

Mean area model	AIC_c_	∆AIC	AIC_ω_	Effect size							
				*t*‐light	*t*‐noise	*t*‐distance	*t*‐light × noise	*t*‐light × distance	*t*‐distance × noise	α	σ^2^
(a) *Anolis* (excluding *A. evermanni*) *N*_male lizards, taxa_ = 265, 13											
L	−9.89	0.00	0.80	−2.39						0.02	<0.001
VN	−4.64	5.25	0.06								
RD + L	−4.31	5.58	0.05								
L + VN	−4.21	5.68	0.05								
RD	−4.16	5.73	0.05								
RD + VN	1.11	11.00	0.00								
L × VN	5.36	15.25	0.00								
RD + L + VN	5.86	15.75	0.00								
RD × L	6.00	15.89	0.00								
RD × VN	8.41	18.30	0.00								
RD + L + VN + L × VN	20.96	30.85	0.00								
RD + L + VN + RD × L	21.34	31.23	0.00								
RD + L + VN + RD × VN	21.35	31.24	0.00								
RD + L + VN + RD × VN + L × VN	46.86	56.75	0.00								
RD + L + VN + RD × L + RD × VN + L × VN	98.37	108.26	0.00								
(b) *Draco* (dewlap) *N*_male lizards, taxa_ = 80, 6											
VN	−7.24	0.00	0.68		0.62					<0.001	<0.001
L × VN	−4.00	3.24	0.13								
L	−3.91	3.32	0.13								
L + VN	−2.38	4.86	0.06								
(c) *Draco* (dewlap + lapel) *N*_male lizards, taxa_ = 80, 6											
L	−5.98	0.00	0.56	−0.56						<0.001	<0.001
VN	−3.85	2.13	0.19								
L + VN	−3.73	2.25	0.18								
L × VN	−1.85	4.12	0.07								

## 4 DISCUSSION

There are an increasing number of studies challenging the proposition that sexual selection will reliably produce hyper‐allometry in ornamentation and other putatively sexually selected morphologies (e.g. Bonduriansky, 2006; Sanchez‐Quiros et al., 2012; South & Arnqvist, 2009; van Lieshout & Elgar, 2009), which can instead often exhibit various other allometric relationships (Bonduriansky, 2007; Bonduriansky & Day, 2003; Eberhard et al., 2018; O'Brien et al., 2018; Voje, 2016). Some of these cases are likely driven by genetic and developmental factors that restrict hyper‐allometries from evolving (Voje & Hansen, 2013; Voje et al., 2014; Pelabon et al., 2013, 2014; but see also Dreyer et al., 2016), but the specific function of display morphologies might also contribute towards isometric or hypo‐allometric scaling patterns (Eberhard et al., 2018; O'Brien et al., 2018; Summers & Ord, 2022). Our findings were clearly consistent with these latter studies. We found no evidence that the large, conspicuous dewlap of male *Anolis* or *Draco* lizards exhibited hyper‐allometry, and despite the *Anolis* dewlap being explicitly used by Kodric‐Brown et al. (2006) to illustrate how sexual selection will lead to static hyper‐allometries in condition‐dependent ornaments. While Kodric‐Brown et al. (2006) focussed on different *Anolis* species to those used in our study (i.e. 17 mainland *Anolis* species taken from Echelle et al., 1978), all species were reported to have static allometric exponents of dewlap area that were clearly greater than 1.5 (i.e. were hyper‐allometric). Furthermore, for the species we examined on Jamaica and Puerto Rico, environmental variables that are likely to be important for signal detection in these lizards seemed to explain much of the variation in isometric and hypo‐static allometries among taxa (Table 1a; Figure 2a,c).

The static exponents of the *Anolis* dewlap appeared to be the product of a complex interplay between the brightness of the environment occupied and the typical distance over which the dewlap was likely to have been viewed by territorial male neighbours (Figure 2a). This finding was largely driven by *Anolis* taxa communicating to distant receivers in darker environments (red line; Figure 2a), which were also lizards that typically possessed the largest dewlaps overall (red dots; Figure 2c). This is intuitive to an extent because larger dewlaps should be more obvious to receivers at greater distances and low light. For example, the exaggeration of other sexual signals seems to have occurred as a function of difficult viewing conditions (e.g. Moseley et al., 2019; Ord et al., 2007; Peters et al., 2007; Ramos & Peters, 2017), which is a predictable adaptive outcome to constraints placed upon signal efficacies (Tazzyman et al., 2014; Wiley, 2017).

What is less intuitive, however, is how the evolution of larger dewlaps to compensate for the difficult viewing conditions of low light to distant conspecific receivers generates shallower and hypo‐allometric scaling patterns (Figure 2a). It implies larger males have developed disproportionally smaller dewlaps for their body size than smaller lizards. This might be expected under a scenario of diminishing returns from investing in the exaggeration of the dewlap beyond a particular absolute size (e.g. Weber's law; Akre et al., 2011; Akre & Johnsen, 2016). That is, once the dewlap has reached a particular large size, there is little added benefit gained in detection with further exaggerating its size and presumably it is costly to do so as well. These costs likely include developmental (e.g. Bolstad et al., 2015; Egset et al., 2011, 2012; Pelabon et al., 2013; Tobler & Nijhout, 2010; see also Dreyer et al., 2016; Knell et al., 2004; Lemaitre et al., 2014; Pelabon et al., 2014; Tidiere et al., 2017) or biomechanical (e.g. Barbosa & Moller, 1999; Basolo & Alcaraz, 2003; Summers & Ord, 2022; Swallow et al., 2000) constraints resulting from the expression of a large morphological structure. This ‘plateauing’ of the allometric relationship resulting in hypo‐allometry in taxa with large (on average) dewlap sizes implies that it is not just the influence of habitat light and receiver distance operating on dewlap size, but additional perceptual, developmental or biomechanical constraints impacting changes in gross dewlap size.

At the opposite end of this complex interaction between light and distance are those taxa typically communicating to conspecific male neighbours over comparatively closer distances. In particular, *A. pulchellus* and *A. sageri* (from Puerto Rico and Jamaica, respectively) occupy bright environments and communicate to conspecifics over short distances and similarly exhibit hypo‐allometries in their dewlap size (Figures 1c and 2a). This, coupled with a generally modest (on average) sized dewlap (Figure 2c), might imply that the dewlap is a less important component of the display repertoire for these species. However, both *A. pulchellus* and *A. sageri* have previously been shown to rely heavily on the dewlap during territorial advertisement display, and more so than many other species (Ord et al., 2013). It would seem, then, that the disproportionately *smaller* dewlaps in larger males compared to small males in these species reflects some other factor. For example, large or conspicuous ornaments and other signal structures likely incur the additional cost of increased predation (Basolo & Wagner, 2004; Ruell et al., 2013; Stuart‐Fox & Ord, 2004; Winandy & Denoel, 2015). Living in open, bright habitats has also been shown to be associated with an increased likely risk of predation for conspicuous males (e.g. Stuart‐Fox et al., 2003), which, in turn, promotes greater selection for cryptic signal strategies in these males (e.g. Stuart‐Fox & Ord, 2004). Perhaps, then, the size of the dewlap in *Anolis* lizards communicating in open, bright habitats has the added associated cost from increased predation, which places a cap on the overall size the dewlap. Predation does appear to influence the evolution of dewlap size in *Draco* lizards, with species living in high‐predation environments tending to have smaller dewlaps (Klomp et al., 2016). However, neither the within (static) or across taxa (evolutionary) allometries of the *Draco* dewlap were explained by habitat type (Figure 2b,d; Tables 1b,c and 2b,c). This is surprising considering both *Draco* and *Anolis* utilize their dewlaps in the same way during territorial display (Ord, Klomp, et al., 2021a).

However, the underlying mechanical morphology of the dewlap differs between *Draco* and *Anolis* (see Ord et al., 2015), which might account for the prominent hypo‐allometry in *Draco* dewlaps with little variation in exponents among taxa (whereas exponents computed for *Anolis* dewlaps were more variable among taxa; Figure 1a,c). The rigid hyoid apparatus that extends the dewlap during display in *Draco* potentially constrains the size of the dewlap. For example, *Draco* species with the largest dewlaps were often observed scraping the end of the dewlap against the substrate. This does not occur in *Anolis* where species with the largest dewlaps rarely touch the substrate because of the way in which the hyoid flexes and stretches as the dewlap opens. This might also explain why *Draco* have evolved the extension to the dewlap in the form of the neck lapel. The extension of the lapel in unison with the dewlap clearly creates a larger combined visual signal (Figure 1b) and presumably enhances the detection of the dewlap by conspecifics. However, the underlying factors contributing to the allometry of the dewlap in *Draco* remain unresolved and provide an exciting opportunity for further study. In particular, the interplay between the underlying hyoid structure that controls the extension of the dewlap, and the extent to which it places a biomechanical cap on dewlap size in *Draco* but not *Anolis*, seems to be a particularly obvious future avenue of research.

More generally, the isometry and hypo‐allometry of the *Anolis* dewlap is surprising given its size appears to be positively correlated with bite force (Lailvaux & Irschick, 2007; Vanhooydonck et al., 2005) and was thought to advertize potential fighting ability to rival males (Curlis et al., 2017; Petelo & Swierk, 2017; Putman et al., 2019; Vanhooydonck et al., 2009). Furthermore, every *Anolis* species in which the allometry of the dewlap has been examined so far has indicated hyper‐allometry (e.g. Echelle et al., 1978; Kodric‐Brown et al., 2006; Petelo & Swierk, 2017). This could purely reflect the manner in which allometries have been previously examined. The standard practice today is to first linearization area values by taking the square‐root to ensure the morphological variable has the same dimensionality as the body size measure (e.g. dewlap size was measured as an area, while body size was measured as a length). All measures are then subject to a natural‐log transform (not a log_10_‐transform; e.g. see recommendations outlined by Bonduriansky, 2007). The majority of allometry data for the *Anolis* dewlap can be sourced to Echelle et al., 1978 who examined 17 mainland species, but did not linearize dewlap area prior to their analyses. All the reported allometric exponents in that study are, therefore, two times greater than if dewlap area had been subject to a square‐root linearization prior to fitting allometry equations. Even still, every species was estimated to have hyper‐allometric exponents ranging from 2.97 to 8.97 (isometry would correspond to a value of two in this instance), which would be equivalent to 1.49 to 2.79 if dewlap area had been linearized. Kodric‐Brown et al. (2006) used Echelle et al.'s (1978) data to support their argument for hyper‐allometry in sexually selected characteristics (NB: Kodric‐Brown et al. (2006) appear to have corrected the original allometric exponents from Echelle et al. (1978) in some way, but how this was done is unclear: their *Anolis* allometric exponents in Figure 1 range from 1.7 to 3.0). Petelo and Swierk (2017) similarly did not linearize dewlap area prior to their analysis, but their estimated exponent again indicated hyper‐allometry in another mainland species (*Anolis aquaticus*: *b* = 3.02, or 1.51 if dewlap area had been linearized).

Our method for estimating dewlap size was biologically comparable to the manner in which the dewlap is viewed by conspecific receivers (i.e. still images of the dewlap fully extended were extracted from videos of free‐living males in mid‐display). In contrast, all previous studies (Echelle et al., 1978; Petelo & Swierk, 2017) have measured dewlap size by manually pulling out the dewlap of captured males using forceps (e.g. see also Lailvaux & Irschick, 2007; Vanhooydonck et al., 2005). The dewlap in *Anolis* is highly elastic (Lailvaux et al., 2015) and can be easily stretched beyond its typical size as it would normally appear to receivers during display (TJO personal observation). We suspect that this practice of manually extending the dewlap by researchers has resulted in non‐linear overestimates of dewlap size. Specifically, larger dewlaps are likely stretched further than smaller dewlaps because larger dewlaps are more elastic, and this results in disproportionately greater overestimates of dewlap size for males with the largest dewlaps; that is hyper‐allometry.

It remains possible that the Jamaican and Puerto Rican *Anolis* species examined in our study have experienced different selection pressures on dewlap size to those species found in mainland locations (i.e. those species examined by Echelle et al. (1978) and Petelo and Swierk (2017)). What these differences might be is unclear. The dewlap is used in a similar fashion across all *Anolis* species as part of a territorial advertisement display (Jenssen, 1977) and is clearly a vital component of social communication in the Jamaican and Puerto Rican species studied here (e.g. Lailvaux & Irschick, 2007; Ord et al., 2013; Vanhooydonck et al., 2005). That is, it seems reasonable to assume that there is a comparable level of positive selection on dewlap size in both island and mainland anole lizards. There are no obvious differences either in the visual environments occupied by mainland and island anoles that might result in differences in signal detection (Fleishman, 1992, 2000). A more obvious explanation might be differences in predation risk (as discussed above), but mainland anoles are generally thought to experience *greater* predation than most island species (Irschick et al., 1997; reviewed by Losos, 2009), which is inconsistent with the observed hyper‐allometry in mainland species and isometry/hypo‐allometry in island species.

Regardless, what is clear is that the putative hyper‐allometry of the *Anolis* dewlap is not evident for Jamaican and Puerto Rican anoles. This is significant because hyper‐allometry has served as evidence for dewlap size being a key target of sexual selection (e.g. Echelle et al., 1978) and been central to the proposition that hyper‐allometries are the hallmark of sexual selection (Kodric‐Brown et al., 2006; see also Green, 1992; Petrie, 1988, 1992). This hypothesis of hyper‐allometry has been so pervasive it is often extended to other systems where sources of sexual selection appear to produce equally extreme hyper‐allometries (sensu Kodric‐Brown et al., 2006). To be clear, our findings do not refute that the dewlap might be used to advertize male condition or potential fighting ability to conspecific receivers. But our results do suggest that the dewlap plays at least an additional role in signal detection, such as acting as a visual amplifier (Candolin, 2003; Gualla et al., 2008; Hasson, 1991; Hebets & Papaj, 2005) to enhance the visibility of the overall territorial display (Charles & Ord, 2012). Deviations from hyper‐allometry in putatively sexually selected characteristics are not unusual, and have been used to question the utility of allometry in determining the likely selection pressures operating on the exaggeration of sexual morphologies (Bonduriansky, 2007; Bonduriansky & Day, 2003; Voje, 2016). The allometries of sexual structures are likely to be particularly vulnerable to confounds of viability selection as well (O'Brien et al., 2017; O'Brien & Boisseau, 2018; Pelabon et al., 2014). Hyper‐allometries are apparent in various animal groups (e.g. Alatalo et al., 1988; Eberhard et al., 2018; Haley & Gray, 2012; Kawano, 2003; Simmons & Tomkins, 1996; Swallow et al., 2005; Tomkins & Simmons, 1996), but function‐specific limitations can nevertheless suppress the evolution of disproportionately larger morphological structures (Voje, 2016; Eberhard et al., 2018; Rodriguez & Eberhard, 2019; Summers & Ord, 2022). Our study suggests that signal efficacy, and the associated conditions in which morphological structures are viewed under, should be added to the list of factors that can influence the allometries of exaggerated sexual structures.

## AUTHOR CONTRIBUTIONS

TCS and TJO conceived the project, designed the study and collected the data. TJO funded the work and obtained ethics and licencing approval to conduct the work. TCS analysed the data and prepared the figures and tables. TCS and TJO wrote the paper.

## CONFLICT OF INTEREST

The authors have no conflict of interest to declare.

### PEER REVIEW

The peer review history for this article is available at https://publons.com/publon/10.1111/jeb.14102.

### OPEN RESEARCH BADGES

Is the author interested in applying for an Open Research Badge?: Yes.

## Supporting information


Figure S1

Figure S2

Tables S1–S3

## Data Availability

Original data on snout‐vent length and dewlap area are provided here: https://doi.org/10.5061/dryad.fttdz08wg Other data on visual noise, ambient light and receiver distance were taken from earlier studies (Ord et al., 2010; Ord, 2012; Ord, Klomp, et al., 2021a) and those data are available here: https://doi.org/10.5061/dryad.1619 https://doi.org/10.5061/dryad.367ns290 https://doi.org/10.5061/dryad.ncjsxksv5 NB: all data can be back transformed to the original raw values with consideration of the particular transforms made that are noted in the methods of this and the original papers. Images and videos used to obtain original data were too large in size and number for hosting on the above repositories and can be obtained directly from TJO through a file share request (t.ord@unsw.edu.au).
