# Supplementary figures and images for: Signal detection shapes ornament allometry in functionally convergent Caribbean Anolis and Southeast Asian Draco lizards
Source: J Evol Biol. 2022 Sep 30;35(11):1508–23. doi: 10.1111/jeb.14102 (PMC9828585; doi:10.1111/jeb.14102)

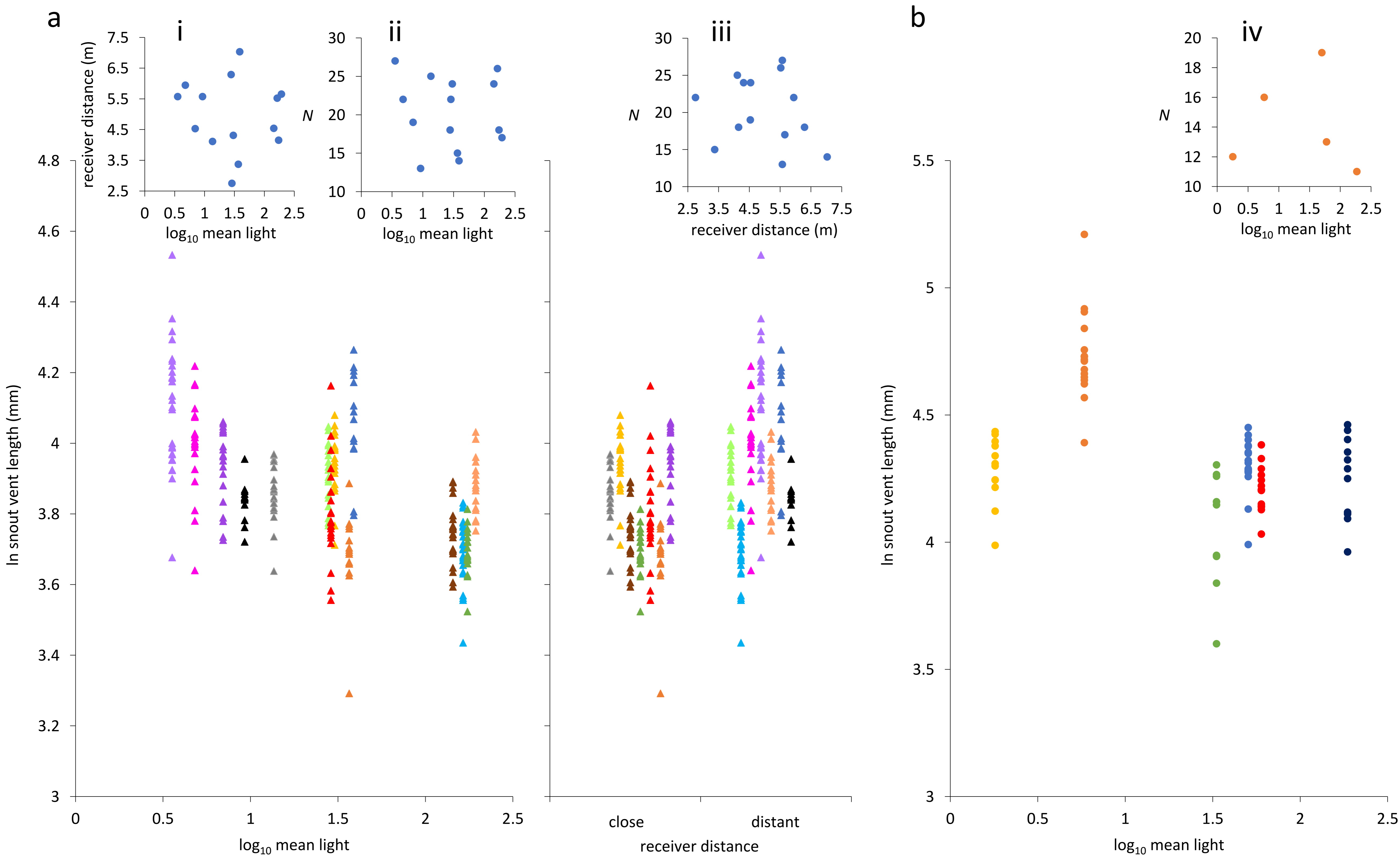

Supplement: Supplementary file 1 — Figure S1 [file JEB-35-1508-s002.pdf]

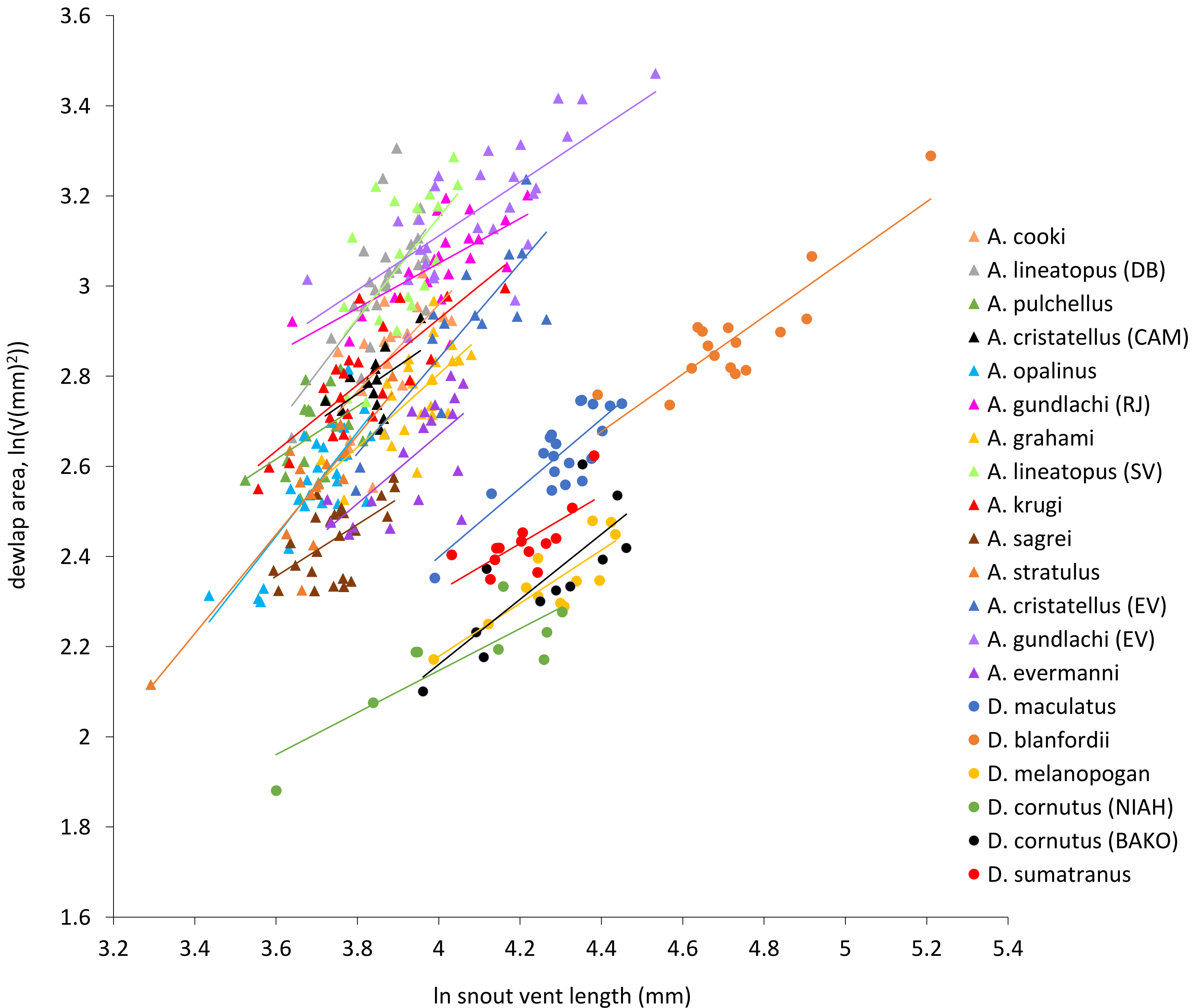

Supplement: Supplementary file 2 — Figure S2 [file JEB-35-1508-s003.pdf]
