# Supplementary material for: Signal detection shapes ornament allometry in functionally convergent Caribbean Anolis and Southeast Asian Draco lizards
Source: J Evol Biol. 2022 Sep 30;35(11):1508–23. doi: 10.1111/jeb.14102 (PMC9828585; doi:10.1111/jeb.14102)
Supplement: Supplementary file 3 — Tables S1–S3 [file JEB-35-1508-s001.docx]

Table S1. Sampling locations, sample size, within lizard repeatability of morphological measures and fit of allometry equations for (a) *Anolis* dewlaps, (b) *Draco* dewlaps and (c) *Draco* dewlap+lapels. Repeatability was computed as *s*_A_^2^/(*s*^2^+ *s*_A_^2^), where *s*_A_^2^ is the variance of mean values across lizards and *s*^2^ is the variance of the three measures taken within a lizard. Values are summarised as a median value, *r*_median_. Note, there was no correlation between sample size and the fit of allometry regressions (p = 0.86, 0.82 and 0.74, respectively).

| Taxon | Location | Sample size (n) | Dewlap area, *r*_median_ | Snout to vent length, *r*_median_ | Allometric coefficient (*R*^2^) |
| --- | --- | --- | --- | --- | --- |
| a) *Anolis* dewlap |  |  |  |  |  |
| *A. cooki* | Bahia de la Ballena, Puerto Rico | 17 | 0.92 | 0.87 | 0.37 |
| *A. cristatellus* (EV) | El Verde, Caribbean National Forest, Puerto Rico | 14 | 0.98 | 0.94 | 0.68 |
| *A. cristatellus* (CAM) | Cambalache Forest, Puerto Rico | 13 | 0.95 | 0.91 | 0.27 |
| *A. evermanni* | El Verde, Caribbean National Forest, Puerto Rico | 19 | 0.96 | 0.98 | 0.47 |
| *A. gundlachi* (EV) | El Verde, Caribbean National Forest, Puerto Rico | 27 | 0.91 | 0.90 | 0.54 |
| *A. gundlachi* (RJ) | Ciales, Puerto Rico | 22 | 0.95 | 0.80 | 0.46 |
| *A. krugi* | El Verde, Caribbean National Forest, Puerto Rico | 22 | 0.96 | 0.90 | 0.64 |
| *A. pulchellus* | El Verde, Caribbean National Forest, Puerto Rico | 18 | 0.97 | 0.85 | 0.19 |

Table S1. continued.

| Taxon | Location | Sample size (n) | Dewlap area, *r*_median_ | Snout to vent length, *r*_median_ | | Allometric coefficient (*R*^2^) | |
| --- | --- | --- | --- | --- | --- | --- | --- |
| *A. stratulus* | El Verde, Caribbean National Forest, Puerto Rico | 15 | 0.92 | 0.81 | | 0.74 | |
| *A. grahami* | Discovery Bay, Jamacia | 24 | 0.85 | | 0.85 | | 0.44 |
| *A. opalinus* | Blue Mountains, Jamacia | 26 | 0.94 | | 0.69 | | 0.64 |
| *A. lineatopus* (DB) | Discovery Bay, Jamacia | 25 | 0.97 | | 0.88 | | 0.37 |
| *A. lineatopus* (SV) | Sun Valley Plantation, Jamacia | 18 | 0.97 | | 0.74 | | 0.37 |
| *A. sageri* | Discovery Bay, Jamacia | 24 | 0.94 | | 0.90 | | 0.33 |

Table S1. continued.

| Taxon | Location | | Sample size (n) | Dewlap area, *r*_median_ | Snout to vent length, *r*_median_ | | Allometric coefficient (*R*^2^) | |
| --- | --- | --- | --- | --- | --- | --- | --- | --- |
| b) *Draco* dewlap |  |  | |  | |  | |  |
| *D. maculatus* | Ulu Gombak, Malaysia | 19 | | 0.86 | | 0.73 | | 0.68 |
| *D. blanfordii* | Ulu Gombak, Malaysia | 16 | | 0.99 | | 0.99 | | 0.76 |
| *D. melanopogon* | Ulu Gombak, Malaysia | 12 | | 0.95 | | 0.98 | | 0.70 |
| *D. cornutus* (NIAH) | Niah National Park, Borneo | 9 | | 0.97 | | 0.97 | | 0.71 |
| *D. cornutus* (BAKO) | Bako National Park, Borneo | 11 | | 0.92 | | 0.96 | | 0.65 |
| *D. sumatranus* | Kota Samarahan, Borneo | 13 | | 0.99 | | 0.98 | | 0.52 |

Table S1. continued.

| c) *Draco* lapel |  |  |  |  |  |
| --- | --- | --- | --- | --- | --- |
| *D. maculatus* | Ulu Gombak, Malaysia | 19 | 0.88 | 0.73 | 0.66 |
| *D. blanfordii* | Ulu Gombak, Malaysia | 15 | 0.99 | 0.99 | 0.79 |
| *D. melanopogon* | Ulu Gombak, Malaysia | 12 | 0.99 | 0.98 | 0.68 |
| *D. cornutus* (NIAH) | Niah National Park, Borneo | 9 | 0.98 | 0.97 | 0.73 |
| *D. cornutus* (BAKO) | Bako National Park, Borneo | 8 | 0.96 | 0.96 | 0.72 |
| *D. sumatranus* | Kota Samarahan, Borneo | 13 | 0.99 | 0.98 | 0.56 |

Table S2. Phylogenetic regressions of *Anolis* dewlap (a) OLS static allometric and (b) evolutionary allometric exponents inclusive *A.* *evermanni* that rank all possible combinations of three key environmental variables: L, ambient light (log_10_); VN, visual noise; and RD, receiver distance.

|  |  |  |  | effect size | | | | | |  | |  |  |
| --- | --- | --- | --- | --- | --- | --- | --- | --- | --- | --- | --- | --- | --- |
| Model | AIC_c_ | ∆AIC | AIC_ω_ | t-light | t-noise | t-distance | t-light x noise | t-light x distance | t-distance x noise | | α | | σ^2^ |
| a) *Anolis* static allometric exponents |  |  |  |  |  |  |  |  |  | |  | |  |
| *N* _male lizards, taxa_ = 284, 14 |  |  |  |  |  |  |  |  |  | |  | |  |
| RD x L | 8.01 | 0.00 | 0.45 | -4.14 |  | -4.33 |  | 4.56 |  | | 0.05 | | <0.001 |
| L | 9.72 | 1.71 | 0.19 | 1.07 |  |  |  |  |  | | 0.26 | | 0.03 |
| RD | 10.50 | 2.49 | 0.13 |  |  |  |  |  |  | |  | |  |
| VN | 10.67 | 2.66 | 0.12 |  |  |  |  |  |  | |  | |  |
| RD + L + VN + RD x L + L x VN | 12.70 | 4.69 | 0.04 |  |  |  |  |  |  | |  | |  |
| RD + L | 14.37 | 6.36 | 0.02 |  |  |  |  |  |  | |  | |  |
| L + VN | 14.61 | 6.60 | 0.02 |  |  |  |  |  |  | |  | |  |
| RD + VN | 15.32 | 7.30 | 0.01 |  |  |  |  |  |  | |  | |  |
| RD + L + VN + RD x L | 16.67 | 8.66 | 0.01 |  |  |  |  |  |  | |  | |  |
| RD x VN | 17.77 | 9.76 | 0.00 |  |  |  |  |  |  | |  | |  |
| RD + L + VN | 20.76 | 12.75 | 0.00 |  |  |  |  |  |  | |  | |  |
| L x VN | 21.03 | 13.01 | 0.00 |  |  |  |  |  |  | |  | |  |
| RD + L + VN + RD x VN | 25.50 | 17.49 | 0.00 |  |  |  |  |  |  | |  | |  |
| RD + L + VN + L x VN | 29.04 | 21.03 | 0.00 |  |  |  |  |  |  | |  | |  |
| RD + L + VN + RD x L + RD x VN + L x VN | 40.98 | 32.97 | 0.00 |  |  |  |  |  |  | |  | |  |

Table S2. continued.

|  |  |  |  | effect size | | | | | | |  |  |  |
| --- | --- | --- | --- | --- | --- | --- | --- | --- | --- | --- | --- | --- | --- |
| Model | AIC_c_ | ∆AIC | AIC_ω_ | t-light | t-noise | t-distance | t-light x noise | t-light x distance | t-distance x noise | α | | | σ^2^ |
| b) *Anolis* taxon mean area |  |  |  |  |  |  |  |  |  |  | | |  |
| *N* _male lizards, taxa_ = 284, 14 |  |  |  |  |  |  |  |  |  |  | | |  |
| L | -8.00 | 0.00 | 0.60 | -1.46 |  |  |  |  |  | 0.01 | | | <0.001 |
| RD | -5.62 | 2.38 | 0.18 |  |  |  |  |  |  |  | | |  |
| VN | -5.57 | 2.43 | 0.18 |  |  |  |  |  |  |  | | |  |
| L + VN | -1.53 | 6.47 | 0.02 |  |  |  |  |  |  |  | | |  |
| RD + VN | 0.61 | 8.61 | 0.01 |  |  |  |  |  |  |  | | |  |
| RD + L | 5.99 | 13.99 | 0.00 |  |  |  |  |  |  |  | | |  |
| RD * L | 5.99 | 13.99 | 0.00 |  |  |  |  |  |  |  | | |  |
| RD + L + VN | 6.25 | 14.25 | 0.00 |  |  |  |  |  |  |  | | |  |
| L x VN | 6.44 | 14.44 | 0.00 |  |  |  |  |  |  |  | | |  |
| RD x VN | 6.84 | 14.84 | 0.00 |  |  |  |  |  |  |  | | |  |
| RD + L + VN + L x VN | 16.67 | 24.67 | 0.00 |  |  |  |  |  |  |  | | |  |
| RD + L + VN + RD x L | 17.84 | 25.84 | 0.00 |  |  |  |  |  |  |  | | |  |
| RD + L + VN + RD x VN | 18.23 | 26.23 | 0.00 |  |  |  |  |  |  |  | | |  |
| RD + L + VN + RD x VN + L x VN | 18.60 | 26.60 | 0.00 |  |  |  |  |  |  |  | | |  |
| RD + L + VN + RD x L + RD x VN + L x VN | 65.03 | 73.03 | 0.00 |  |  |  |  |  |  |  | | |  |

Table S3. Phylogenetic regressions of (a) *Anolis* and (b) *Draco* intercept values of dewlap area taken from OLS static allometry analyses in which body size was centred on the global mean of all species. Models rank all possible combinations of three key environmental variables: L, ambient light (log_10_); VN, visual noise; and RD, receiver distance. Findings are generally consistent with those reported in Table 2. Visual inspection of plots for light and visual noise in (b) did not reveal a credible association for these variables.

|  |  |  |  | effect size | | | | | |  |  |  |
| --- | --- | --- | --- | --- | --- | --- | --- | --- | --- | --- | --- | --- |
| Mean area model | AIC_c_ | ∆AIC | AIC_ω_ | t-light | t-noise | t-distance | t-light x noise | t-light x distance | t-distance x noise | α | | σ^2^ |
| a) *Anolis* (excluding *A. evermanni*) |  |  |  |  |  |  |  |  |  |  | |  |
| *N* _male lizards, taxa_ = 265, 13 |  |  |  |  |  |  |  |  |  |  | |  |
| L | -17.68 | 0.00 | 0.59 | -3.13 |  |  |  |  |  | 0.03 | | <0.001 |
| L+RD | -15.41 | 2.28 | 0.19 |  |  |  |  |  |  |  | |  |
| L+VN | -14.64 | 3.04 | 0.13 |  |  |  |  |  |  |  | |  |
| L+RD+VN | -11.24 | 6.44 | 0.02 |  |  |  |  |  |  |  | |  |
| L*RD | -11.14 | 6.54 | 0.02 |  |  |  |  |  |  |  | |  |
| L*VN | -10.44 | 7.24 | 0.02 |  |  |  |  |  |  |  | |  |
| VN | -10.19 | 7.49 | 0.01 |  |  |  |  |  |  |  | |  |
| RD | -10.11 | 7.57 | 0.01 |  |  |  |  |  |  |  | |  |
| RD+VN | -7.51 | 10.17 | 0.00 |  |  |  |  |  |  |  | |  |
| L*RD+VN | -6.17 | 11.52 | 0.00 |  |  |  |  |  |  |  | |  |
| L*VN+RD | -5.88 | 11.80 | 0.00 |  |  |  |  |  |  |  | |  |
| L+RD*VN | -5.73 | 11.95 | 0.00 |  |  |  |  |  |  |  | |  |
| L*RD*VN | -5.68 | 12.00 | 0.00 |  |  |  |  |  |  |  | |  |
| RD*VN | -4.52 | 13.16 | 0.00 |  |  |  |  |  |  |  | |  |

Table S3. continued.

|  |  |  |  | effect size | | | | | |  |  |  |
| --- | --- | --- | --- | --- | --- | --- | --- | --- | --- | --- | --- | --- |
| Mean area model | AIC_c_ | ∆AIC | AIC_ω_ | t-light | t-noise | t-distance | t-light x noise | t-light x distance | t-distance x noise | α | | σ^2^ |
| b) *Draco* (dewlap) |  |  |  |  |  |  |  |  |  |  | |  |
| *N* _male lizards, taxa_ = 80, 6 |  |  |  |  |  |  |  |  |  |  | |  |
| L | 1.35 | 0.00 | 0.53 | 2.91 |  |  |  |  |  | <0.001 | | <0.001 |
| VN | 1.60 | 0.24 | 0.47 | 2.82 |  |  |  |  |  | <0.001 | | <0.001 |
| L+VN | 11.16 | 9.80 | 0.00 |  |  |  |  |  |  |  | |  |
| L*VN | 40.96 | 39.61 | 0.00 |  |  |  |  |  |  |  | |  |
